# Identification of Distribution Characteristics and Epidemic Trends of Hepatitis E in Zhejiang Province, China from 2007 to 2012

**DOI:** 10.1038/srep25407

**Published:** 2016-05-05

**Authors:** Kui Liu, Jian Cai, Shan Wang, Zhaofan Wu, Li Li, Tao Jiang, Bin Chen, Gaofeng Cai, Zhenggang Jiang, Yongdi Chen, Zhengting Wang, Xuhui Zhu, Liuru Hu, Hua Gu, Jianmin Jiang

**Affiliations:** 1 Department of Science Research and Information Management, Zhejiang Provincial Center for Disease Control and Prevention, Hangzhou, Zhejiang Province, People’s Republic of China; 2 Department of Infectious Diseases Control and Prevention, Zhejiang Provincial Center for Disease Control and Prevention, Hangzhou, Zhejiang Province, People’s Republic of China; 3 Department of Epidemiology & Health Statistics, Zhejiang University, Hangzhou, Zhejiang Province, People’s Republic of China; 4 Department of Epidemiology & Health Statistics, Fudan University, Shanghai, People’s Republic of China; 5 Nine Squadrons of First Terrain Metering, State Bureau of Surveying and Mapping, Xi’an City, Shaanxi Province, People’s Republic of China

## Abstract

Hepatitis E virus is a common hepatotropic virus that causes serious gastrointestinal symptoms. Data of reported HEV cases in Zhejiang Province was collected between 2007 and 2012. Descriptive epidemiological methods and spatial-temporal epidemiological methods were used to investigate the epidemiological trends and identify high-risk regions of hepatitis E infection. In this study, the average morbidity of hepatitis E infection was 4.03 per 100,000 in Zhejiang Province, peaking in winter and spring. The ratio between the male and the female was 2.39:1, and the high-risk population was found to be aged between 40 and 60. Trend surface analysis and IDW maps revealed higher incidences in the northwestern counties. The spatial-temporal analysis showed comparable incidences in the counties at the basins of three rivers, mostly under administration of Hangzhou Municipality. Besides, the seasonal exponential smoothing method was determined as the better model for the retrieved data. The epidemiological characteristics of HEV suggested the need of strengthened supervision and surveillance of sanitary water, sewage treatment and food in high-risk areas especially around the Spring Festival. Additionally, time series model could be useful for forecasting the epidemics of HEV in future. All these findings may contribute to the prevention and control of HEV epidemics.

Hepatitis E, an acute live infection caused by hepatitis E virus (HEV), is one of the most common epidemic diseases throughout Asia and Africa^1^. It is mainly transmitted via fecal contamination of the oral cavity. The known animal reservoirs of HEV include wild boars, swine, mongoose, deer, rabbits and camels^2^,^3^,^4^,^5^,^6^. The available evidence shows that HEV strains from animal reservoirs are close to human pathogenic HEV strains and several reports evidenced transmission from swine to humans through the consumption of uncooked sausage^7^,^8^,^9^. Similar to the clinical manifestations of hepatitis A, patients with acute HEV infection mainly present jaundice and the pain of right upper quadrant accompanied by fever, nausea, vomiting, asthenia, and joint pain^10^,^11^,^12^. HEV was identified as self-limited without progression to chronicity^13^,^14^. However, increasing evidence indicates that immunocompromised patients coupled with HEV infection may progress to the prognosis of the chronic hepatitis and cirrhosis^15^,^16^,^17^. The epidemiology of HEV infection finds its mortality rates ranging from 0.2% to 4.0%^18^. Higher mortality rates have been reported among the infants under 2 years of age and pregnant women^19^,^20^, and the middle-aged and elderly men may be more susceptible to symptomatic HEV infection^21^,^22^. Additionally, more autochthonous cases of HEV are being recognized in the developed countries such as UK and France, but this increase is not as large as seen in the epidemics that occur in the developing countries^23^,^24^.

HEV aroused huge attention as a significant public health issue in China in the past decade^25^. Thanks to improved sanitation and living environment, hepatitis E is under better control with new infections coming primarily from sporadic cases and occasional food-borne outbreaks^26^. However, its morbidity in economically developed Zhejiang Province was found to be high in 2011, and the specific transmission route was still controversial.

This study aimed to identify HEV epidemic features using techniques in spatial-temporal analysis. Geographic information system (GIS) showed vital advantages over the conventional epidemiological techniques in the primitive surveillance of communicable diseases, rapid quantification of the susceptible population, effective allocation of the health resource and timely formulation of the prevention strategies. The spatial differentiation of the HEV infection provided a map visualizing the diversity of HEV infection in the different regions, and the in-depth statistics with spatial autocorrection could recognize the graphical clusters of HEV infection, which is helpful in identifying the potential risk factors and evaluating the efficiency of intervention measures^27^. In our study, time series models were also chosen to provide scientific clues for the control and intervention with HEV infection in the Province. Autoregressive integrated moving average (ARIMA) model and Exponential Smoothing Method (ESM) were compared for the better one by error testing indexes. ARIMA model, widely used in non-stationary time series, can take into account the changing trends, periodic changes, and random disturbances, removing seasonal patterns^28^. For instance, FY Tang *et al.* established an ARIMA model based on the shigella cases of Jiangsu Province from 2001 to 2011 to predict the potential cases from August to December in 2011, and the prediction was almost identical to the observed data^28^. ESM can also be used for the medium- and long-term prediction^29^.

To our knowledge, this study is the first to identify the characteristics of HEV epidemics in Zhejiang Province using the spatial-temporal statistic methods. The results would be helpful in detecting the potentially high-risk regions for necessary interventions.

## Materials and Methods

### Ethics Statement

This study was approved by the Ethics Committee of Zhejiang Provincial Center for Disease Control and Prevention. All personal information was kept confidential as required.

### Profile of Zhejiang Province

Zhejiang Province, an economically developed Province, is located in the southeast China between longitudes 118°E-123°E and latitudes 27°N–32°N. It has a land area of 101,800 square kilometers, accounting for 1.06% of the whole country and making itself one of China’s smallest Provinces. It is composed of two sub-provincial cities (Hangzhou and Ningbo) and nine prefecture-level cities (Wenzhou, Huzhou, Jiaxing, Shaoxing, Jinhua, Quzhou, Zhoushan, Taizhou and Lishui) covering 90 counties. As a coastal Province, Zhejiang features a subtropical monsoon climate with the annual mean temperature between 16.3 and 18.4 °C and the annual precipitation ranging from 1489.5 to 1903.3 mm in 2012.

### Data Collection

In China, uniform diagnostic criteria are set for notifiable diseases. Hospitalized patients diagnosed as hepatitis E should be immediately reported through China information system of disease prevention and control by local physicians. Data of HEV cases in Zhejiang Province from 2007 to 2013 was exported from this system^30^. Two staff members (Xuhui Zhu and Zhaofan Wu) independently extracted vital details of the cases as of the day of onset, including the year of the report and the patient gender, age, address and occupation. The population data was also retrieved from the same system.

### Analysis of Epidemiological Characteristics

Effective data was abstracted from the exported documents, and the distribution and epidemiological characteristics were depicted for age, gender, season, and occupation.

### IDW for Interpolation Maps

The inverse distance weighted (IDW) interpolation was employed to predict the incidences of hepatitis E at the individual geographic spots under county level^31^. The results of IDW interpolation generated maps with estimated HEV infections from 2007 to 2012.

### Spatial Variation Analysis

Spatial analysis was aimed to detect geographic variation in relation to HEV outbreak and to explore the potential clustering regions^32^. Firstly, the trend surface analysis was done to frame the overall tendencies and to identify the outliers of the HEV incidence in different geographical locations^33^. Z is the dependent variable (incidence rates), and X and Y are independent variables^28^. Autocorrelation analysis was carried out, including general spatial autocorrelation analysis and local spatial autocorrelation analysis. The general autocorrelation used the Global Moran’s I Index^34^. Moran’s I was defined as follows:


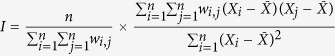


where n was the number of counties, *X*_*i*_ and *X*_*j*_ were the indicators of autocorrelations from unit index i and j. *W*_*i,j*_ was the matrix of spatial weights. If unit i was adjoined to unit j, *W*_*i,j*_ = 1; if not, *W*_*i,j*_ = 0. Moran’s I Index was from −1 to 1. Moran’s I > 0 implied clustering in the spatial distribution, Moran’s Index < 0 dispersing in the spatial distribution, and Moran’s Index = 0 a random spatial distribution. If the *P* value of Global Moran’s Index was statistically significant, the local autocorrelation analysis of Local Moran’s I and Local Getis-Ord G were both done to determine the positive autocorrelation (High-High or Low-Low autocorrelation) or negative autocorrelation (High-Low or Low-High autocorrelation), thus revealing hotspots^35^,^36^,^37^.

### Pure Temporal Clustering Analysis and Spatio-temporal Analysis

Discrete Poisson model to identify temporal clustering of infectious HEV cases was performed by SaTScan software^37^. Kulldorff’s space-time scan statistics was also done with SaTScan software to recognize the clusters and the year of clustering^38^. A cylindrical window scanning across Zhejiang Province revealed the time and regions of clustering, the base diameter of this moving window representing the potential areas of clustering and the height representing the time of clustering^39^. In our study, the maximum spatial cluster size and maximum temporal cluster size were all set to 50%. Log-likelihood Ratio (LLR) was employed to identify the special clusters by comparing the observed incidence with expected one^40^. Eventually, Monte Carlo test was conducted to determine the most likely clusters^41^.

### Time-series Analysis

Exponential smoothing method (ESM), a frequently used time series model, can be utilized for the short-term prediction and the resolution of the problem involving medium- and long-term prediction^42^,^43^. In ESM, an initial value in prediction period was determined as the average of the values in the first few periods. With this new observed value appeared, the earliest one was removed and the new observed value entered in the group. Thus, each new prediction value was calculated based on the new observed value, the initial predicted value and weight of the latest observed value^44^. Another widely used time series model is ARIMA (p, d, q), consisting of three sections in the order of auto regression (p), the degree of difference (d) and the order of moving average (q)^45^. If the original data was not smooth, the moderate finite difference and/or exponential transform would be implemented to transform data into the stationary one^46^. Both autocorrelation function (ACF) and partial autocorrelation function (PACF) were examined to confirm the optimal parameters of p and q. Finally, the Lung-Box tests for white noise test and Bayesian Information Criterion (BIC) for the optimal goodness-of-fit were performed to decide on the ultimate model^47^,^48^. SPSS was used for the establishment of the optimal model of ESM and SAS for that of ARIMA. A better model was ultimately determined by minor error testing indexes, including Mean Absolute Error (MAE), Mean Absolute Percentage Error (MAPE), Mean Square Error (MSE) and Root Mean Square Error (RMSE), which can depict system errors^49^. Ultimately, compared with the actual HEV reported data in 2013, the predicted value (predicted cases with 95% confidence interval) were calculated through the optimal model and the predicted accuracy rate was also evaluated.

### Statistical Analysis

Software used for spatial analysis included the ArcGIS software (version 10.1, SERI Inc.; Redlands, CA, USA) and SaTScan (version 9.1.1, Boston, MA, USA). The time series model was determined using SAS 9.2 (SAS Institute Inc., Cary, NC) and SPSS Statistics 20.0 (SPSS Inc, Chicago, USA). All the results were considered statistically significant if *P* < 0.05 with two sides.

## Results

### Epidemiological Characteristics

**Figure 1:**
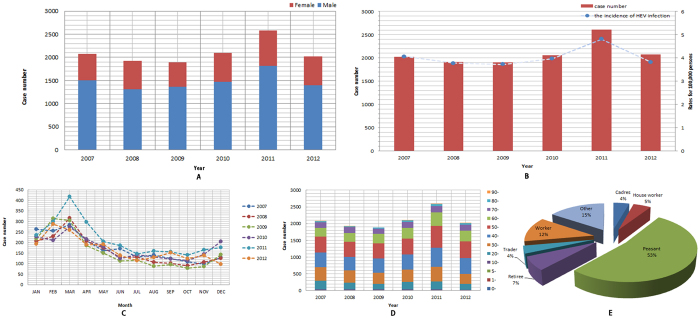
Epidemiological characteristics of HEV infection in Zhejiang Province, China from 2007–2012. (**A**) Gender distribution of HEV cases. (**B**) Incidence rate of HEV infection. (**C**) Monthly distribution of HEV cases. (**D**) Aged distribution of HEV cases. (**E**) Occupational distribution of HEV cases.

Excluding cases with unclear addresses, we screened out a total of 12582 (8869 male and 3713 female with a sex ratio of 2.39:1) cases in Zhejiang Province from 2007 to 2012 from the system above. The incidence averaged 4.03 per 100,000 in the 5 years with 4.06 per 100,000 in 2007, 3.77 per 100,000 in 2008, 3.73 per 100,000 in 2009, 3.97 per 100,000 in 2010, 4.82 per 100,000 in 2011 and 3.81 per 100,000 in 2012. Peaking in March, the epidemics generally clustered between January and April at the turn of winter. Besides, the age distribution of the selected population ranged from 1 to 98 years old, with the high-risk ages being mainly between 40 and 60. Occupational distribution of all reported cases was, from highest to the lowest, peasants (53%), and workers (12%), retirees (7%), house workers (5%), cadres (4%), traders (4%) and others (15%). All above were shown in Fig. 1. Further analysis was made to identify the differences in relation to gender and occupation (Pearson χ^2^_(5)_ = 432.1290, *P* < 0.001). The results showed that males were more susceptible to HEV infection than females except house workers (Table 1).

### Incidence Maps

**Figure 2:**
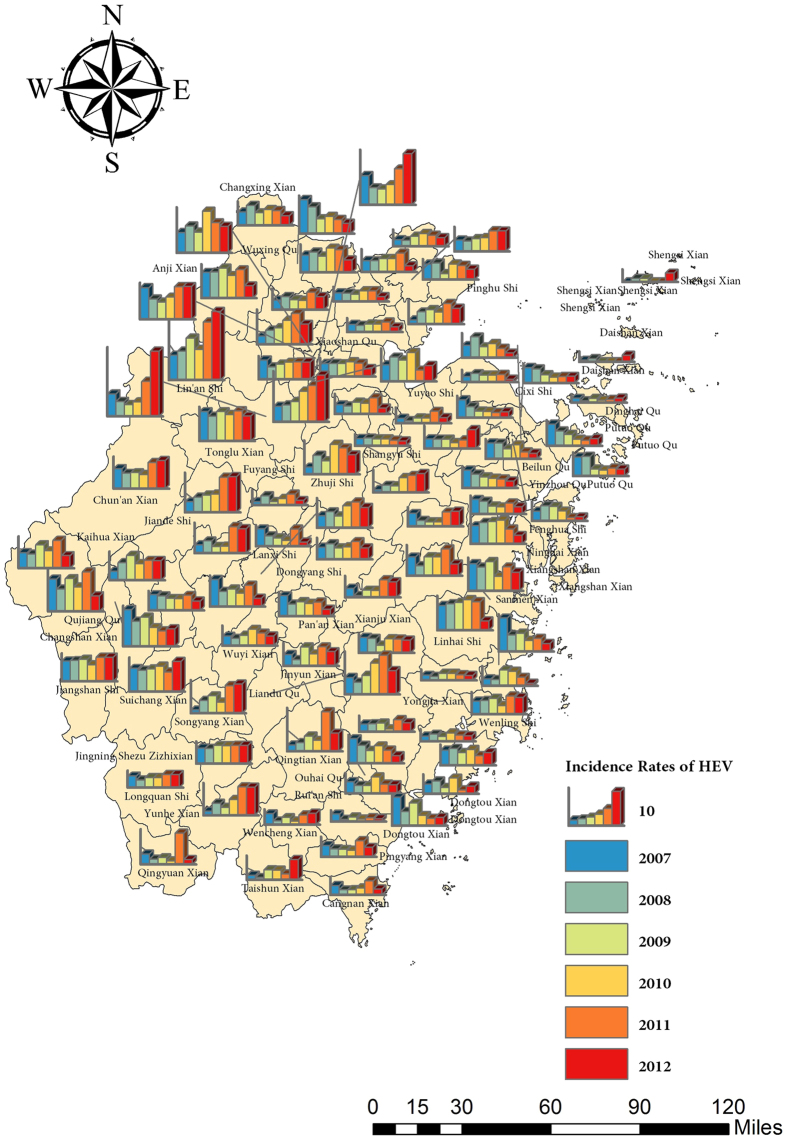
Incidence map of HEV infection in Zhejiang Province from 2007 to 2012. This map was created by ArcGIS software (version 10.1, ESRI Inc.; Redlands, CA, USA). Homepage of ArcGIS software was https://www.esri.com/.

**Figure 3:**
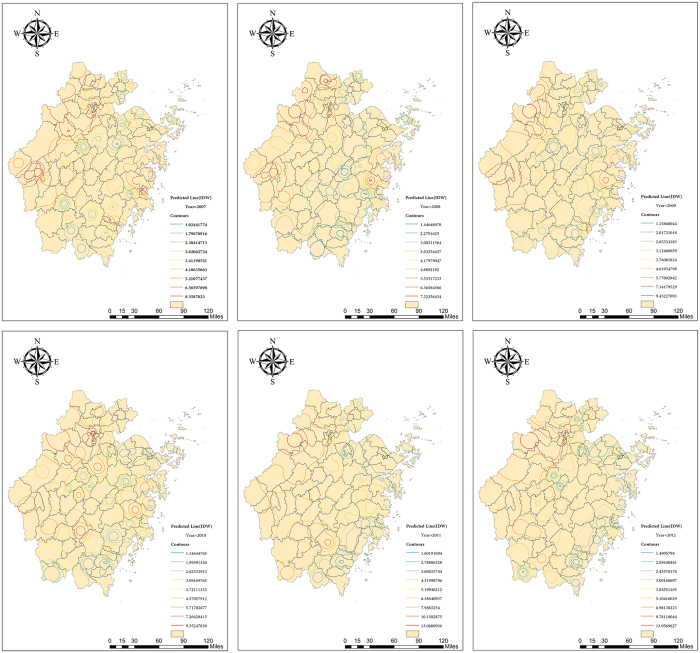
IDW interpolation of HEV Infection map in Zhejiang Province from 2007 to 2012. This map was created by ArcGIS software (version 10.1, ESRI Inc.; Redlands, CA, USA). Homepage of ArcGIS software was https://www.esri.com/.

The incidences of HEV infection from 2007–2012 in Zhejiang Province were mapped (Fig. 2), and the IDW interpolation was performed for the prediction of all the incidences in the map (Fig. 3). The high-risk regions were found to be in northwest and eastern coastal areas of Zhejiang Province in 2007. Afterwards, the incidence rates both in the northwest corner and eastern coastal areas shrank gradually by 2012. However, it is worth mentioning that the incidence in 2011 presented the highest contour line in northwest areas, suggesting clusters there. Besides, comparable incidence rates were observed in the northwest counties whereas low incidence rates in other area.

### Trend Surface Analysis

**Figure 4:**
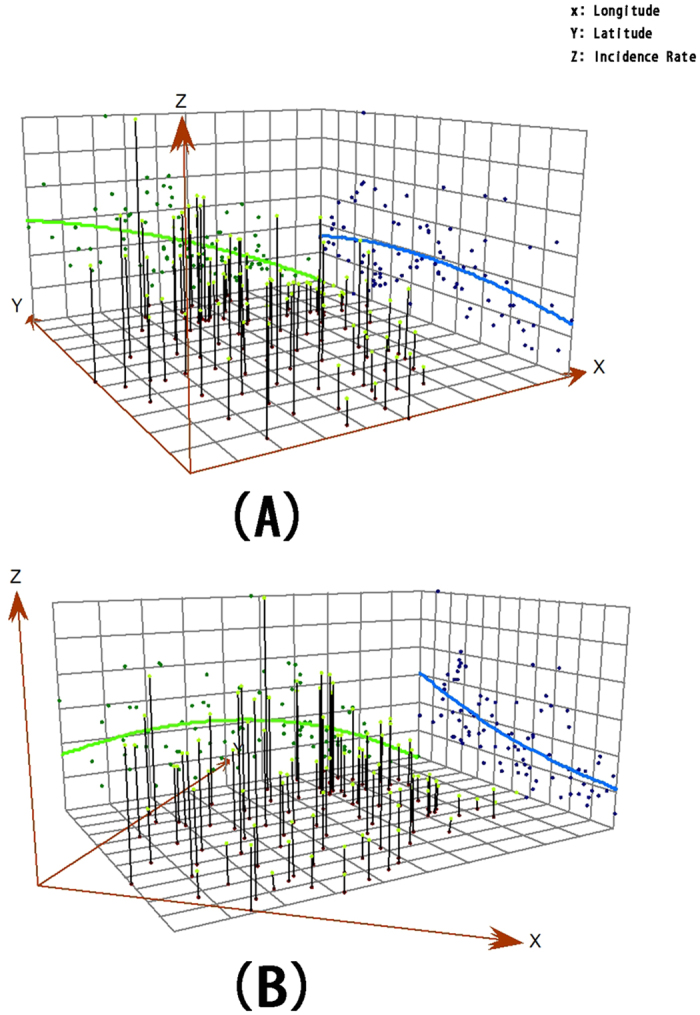
Trend surface analysis of HEV incidence in Zhejiang Province in 2011. This map was created by ArcGIS software (version 10.1, ESRI Inc.; Redlands, CA, USA). Homepage of ArcGIS software was https://www.esri.com/.

The trend surface analysis was employed to identify the geographic trends of the incidence (Fig. 4). A coordinate system was created (one axis for each direction with X for West-East and Y for South-North). The projections of incidence rates (Z axis) reflected the variation trend of West-East with South-North and Southwest-Northeast with Southeast-Northwest. The trend surface analysis implied a higher incidence of HEV infection in the northwest Zhejiang Province (Table 2).

### Spatial Distribution

In the spatial clustering analysis, the general autocorrelation suggested that HEV infected cases were not of random distribution (Table 3). Results of local Moran’s I and local Getis-Ord G showed 29 high-high clusters, 8 low-low clusters and 1 low-high cluster at the county level (Table 4). Lin’an City was found to have the high-high clusters for the longest period of time (5 years), followed by Xihu County (3 years), Fuyang City (2 years), Gongshu County (2 years), Shangcheng County (2 years), Tonglu County (2 years), Xiacheng County (2 years), and Yuhang County (2 years). These areas are all under the administration of Hangzhou Municipality, which means that 21 high-high clusters (72.4%) of HEV infection were located in the provincial capital.

### Temporal Clustering Analysis and Spatio-temporal Analysis

**Figure 5:**
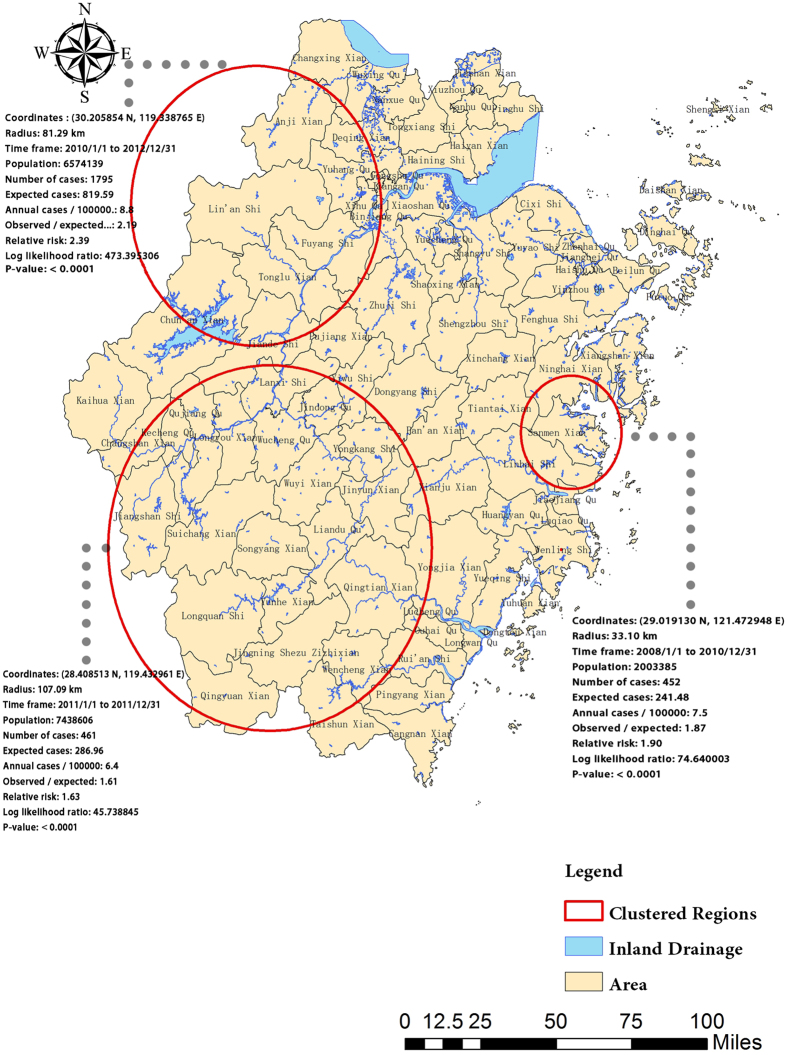
Spatial-temporal clustering of HEV infection from 2007 to 2012 in Zhejiang Province. The most likely cluster included Lin’an, Tonglu, Anji, Fuyang, Yuhang, Xihu, Gongshu, Deqing, Chun’an, Shangcheng, Jiande, Binjiang, Xiacheng; The secord-most likely cluster included Sanmen, Linhai, Ninghai; The third-most likely cluster included Songyang, Yunhe, Suichang, Liandu, Wuyi, Longquan, Jingning,Wucheng, Longyou, Qingtian, Qujiang, Jinyun, Jiangshan, Yongkang, Wencheng, Jindong, Kecheng, Qingyuan, Lanxi, Taishun, Changshan. This map was created by ArcGIS software with Homepage of https://www.esri.com/ (version 10.1, ESRI Inc.; Redlands, CA, USA) and SaTScan software (version 9.1.1, Boston, MA, USA). SaTScan TM is a trademark of Martin Kulldorff. The SaTScan TM software was developed under the joint auspices of (i) Martin Kulldorff, (ii) the National Cancer Institute, and (iii) Farzad Mostashari of the New York City Department of Health and Mental Hygiene.

In the temporal clustering analysis, the discrete Poisson model found the vital temporal clusters in the year of 2011. Besides, the combined spatio-temporal analysis showed three clustering regions from 2007 to 2012 (Fig. 5).

### Time-series analysis

**Figure 6:**
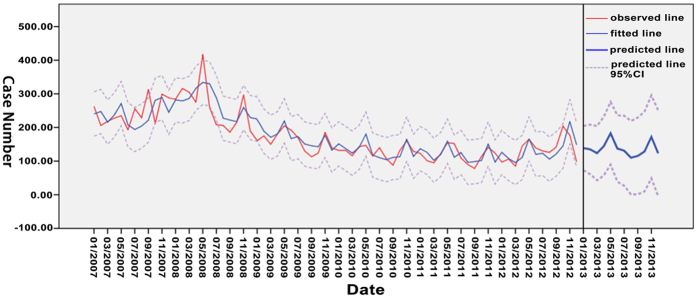
Prediction of time series model for the HEV infection of Zhejiang Province in 2013 using the simple seasonal ESM. This map was created by SPSS Statistics 20.0 (SPSS Inc, Chicago, USA).

The optimal time series models were created using SAS and SPSS, including simple seasonal ESM and ARIMA (6,1,0). Further screening was done based on the indices of MAE, MAPE, MSE, and RMSE. Simple seasonal ESM was finalized as the ultimate model (Table 5 and Fig. 6). Additionally, the predicted accuracy rate of simple seasonal ESM was 75% (9/12), as were shown in Table 6.

## Discussion

This study was aimed to identify the epidemiological characteristics, the spatial-temporal trend, and the regular pattern of HEV infection in Zhejiang Province. The findings showed that men were more susceptible to HEV infection than women probably because men had more chances to contact HEV due to underlying occupational exposure. The epidemiological characteristics of time distribution suggested that the epidemics frequently clustered from January to April, during which time falls the Spring Festival in China. This is when the enormous population flow occurs and activities of visiting relatives and friends surge, a phenomenon that increases difficulty in public health surveillance, particularly in the supervision of drinking water and food. This warrants strengthened monitoring of drinking water and food and health education in hand washing during this period of time. Also, two major prevention strategies, reducing exposure to HEV and inducing autoimmunity by vaccination, have been globally proven effective in the prevention and control of HEV infection^50^,^51^. HEV vaccine, with increasing maturity of the clinical application, should be considered for an annual immunization plan in specific population before January in China. Additionally, different from the age ranged from 20 to 40 of the high risk population in the previous study, vulnerable population was found to be aged between 40 and 60 in our study^52^. Accordingly, the target population for health education and vaccination strategy should be adjusted.

ArcGIS and Scan statistics have been widely applied to detect the abnormal patterns of temporal and spatial clusters in the epidemiological studies^41^,^53^,^54^,^55^. In the present study, the incidence maps with IDW revealed a higher incidence of HEV infection in the northwest counties from 2007 to 2012, which agreed with the findings of the trend surface analysis. The significant clustering pattern of the HEV epidemics from 2007 to 2012 was firstly proved by the general spatial autocorrelation statistics. Then, a total of 29 high-high clusters were discerned by local autocorrelation analysis in spatial dimension. Thus, based on the findings of clusters, this identified locations should pay more attention to the prevention and control of HEV infection and increased available clinical resources allocation in high-risk locations. Interestingly, nearly 73 percentages of high-high clusters were located in the administrative areas of Hangzhou Municipality in the northwest of Zhejiang Province. The Spatio-temporal analysis identified three clusters, the most likely cluster of which was also in Hangzhou with a relative risk of 2.39. We suspected that such a distribution pattern might have been attributed to the lack of clean water sources and inadequate sewage system. To confirm our hypothesis and further explore contributing factors, we investigated the main rivers in these clustering areas. We found that these clusters were laced with three inland drainages: Qiantang River, Jiao River and Ou River. Meanwhile, Lin’an City was the husbandry base of sheep and cow, and Yuhang County served as the main pork and poultry base in Hangzhou^56^. Therefore, the inadequate sewage system and the improper disposition of animal wastes in these areas might have contributed to the HEV infection along Qiantang River, partly explaining the high incidence in Hangzhou. Additionally, Jiao River and Ou River, both located at the estuary of the East Ocean, have abundant fishing resources, and some infected marine products such as oyster might lead to the epidemics in these clustering areas. Although in-depth field investigations were not performed to verify our hypothesis, the findings could help with control and prevention of HEV infection in Zhejiang Province.

With the diverse mechanisms and preconditions, each model has its own limitations in forecasting epidemics. With the obvious seasonal trend of the HEV infection, the time series models were created. Both models fit our data well, and the simple season ESM was chosen as the optimal time series because of lesser errors. Besides, the actual number of reported cases in 2013 further validated its reliability. These findings could provide clues for the model selection in the prediction of HEV infection.

### Limitations

Several limitations should be mentioned with this study. 1) Some potential carriers or asymptomatic cases might not have been captured in available system and retrospective study itself, which might have led to bias. Besides, anti-HEV detection in large country was more easily available and/or used than rural area. Hence, the potential phenomenon of clusters might be not only related to the epidemic but the capacity of detection; 2) Local Moran’s I could reflect the seriousness of the epidemic in spatial pattern, but it might be disturbed by population fluctuation; the power of circular scan statistics could be limited in some irregular counties; 3) Due to inadequacy in risk factors and geographic, climatic and socioeconomic information, a more intensive study was impossible with the correlation analysis and regression model; 4) Some hypotheses have not been verified because further field epidemiologic investigations were not carried out; 5) With only two models being tested in our study, some better models might been detected among the artificial neural network model, Markov model and other models.

## Conclusions

This study investigated the epidemiological characteristics, spatial distribution, temporal distribution and spatial-temporal distribution of HEV infection in Zhejiang Province from 2007 to 2012. The disease tended to cluster from January to April at the turn of winter, suggesting an underlying seasonal variation. The age range of the susceptible population was found to be 40–60 against the previously reported 20–40. Geographically, HEV infection scattered across the Province and clustering was mainly obvious in the northwest areas. The spatial distribution analysis and spatial-temporal distribution analysis identified a total of 29 high-high spots in three different clusters, and all were associated with river systems. Thus, strengthening food and water supervision was critical in controlling and preventing the epidemics particularly around the Spring Festival. The seasonal ESM fit better than ARIMA model in forecasting the reported epidemics of HEV infection. Despite its limitations, this study may contribute to the allocation of the health resources, surveillance of high-risk regions, population immunization, and identification of possible influential factors.

## Additional Information

**How to cite this article**: Liu, K. *et al.* Identification of Distribution Characteristics and Epidemic Trends of Hepatitis E in Zhejiang Province, China from 2007 to 2012. *Sci. Rep.* **6**, 25407; doi: 10.1038/srep25407 (2016).

## Figures and Tables

**Table 1:** Differences in gender and occupation among HEV infected population.

Gender	Occupations					
	Peasant	House worker	Cadres	Worker	Trader	Retiree
Male	4796	213	429	1151	338	547
Female	1820	401	119	386	130	292

**Table 2:** Fluctuations in HEV incidence in Zhejiang Province from 2007 to 2012.

Year	Trend from West-East	Trend from South-North	Trend from Southwest-Northeast	Trend from Southeast-Northwest
2007	Decrease	Inversed U Shape	Inversed U Shape	Increase
2008	Decrease	Increase	Inversed U Shape	Increase
2009	Decrease	Inversed U Shape	Decrease	Increase
2010	Decrease	Increase	Inversed U Shape	Increase
2011	Decrease	Inversed U Shape	Decrease	Increase
2012	Decrease	Increase	Inversed U Shape	Increase
Total	Decrease	Increase	Inversed U Shape	Increase

**Table 3:** General spatial autocorrelation of HEV infection in Zhejiang Province by Global Moran’s I.

Year	Moran’s I Index	Moran’s I Z-score	Moran’s I*P*-value
2007	0.143860	2.129288	0.033230
2008	0.261897	3.740526	0.000184
2009	0.308836	4.428792	0.000009
2010	0.504629	7.111626	0.000000
2011	0.418085	5.929628	0.000000
2012	0.512331	7.461609	0.000000
Total	0.549637	7.764210	0.000000

**Table 4:** Local autocorrelation analysis of HEV in Zhejiang Province by Local Moran’s I and Local Getis-Ord G.

Year	Area	*LG*_*i*_Z score	*LGi* P value	*LM*_*i*_ Index	*LM*_*i*_ Z score	*LM*_*i*_ P value	Correlation Type	Number of Reported cases	Geographic Size (square kilometre)
2007	Changshan	2.323365	0.020160	0.000278	3.976237	0.000070	High-High Cluster	27	1091.47
2007	Sanmen	2.761487	0.005754	0.000208	3.459347	0.000541	High-High Cluster	27	990.587
2008	Lin’an	2.874604	0.004045	0.000096	3.994173	0.000065	High-High Cluster	46	3108.66
2008	Wencheng	−2.735206	0.006234	0.000218	3.445379	0.000570	Low-Low Cluster	3	1270.68
2008	Daishan	−2.742113	0.006105	0.000136	3.421568	0.000623	Low-Low Cluster	2	268.933
2009	Lin’an	4.155629	0.000032	0.000189	7.939035	0.000000	High-High Cluster	68	3108.66
2009	Kaihua	3.439441	0.000583	0.000178	4.247612	0.000022	High-High Cluster	20	2243.45
2009	Jiangshan	2.859664	0.004241	0.000106	3.0258	0.002480	High-High Cluster	33	2000.25
2009	Changshan	3.906149	0.000094	0.000557	8.004969	0.000000	High-High Cluster	28	1091.47
2009	Kecheng	3.301447	0.000962	0.000549	5.515519	0.000000	High-High Cluster	27	620.122
2009	Qujiang	3.277453	0.001047	0.000409	4.133326	0.000036	High-High Cluster	37	1741.49
2009	Sanmen	2.937537	0.003308	0.000290	4.858313	0.000001	High-High Cluster	30	990.587
2010	Lin’an	2.598504	0.009363	0.000079	3.305329	0.000949	High-High Cluster	50	3108.66
2010	Xiacheng	2.882534	0.003945	0.001347	4.392129	0.000011	High-High Cluster	30	30.5015
2010	Shangcheng	2.520364	0.011723	0.000837	2.884501	0.003920	High-High Cluster	23	26.3608
2010	Gongshu	2.882534	0.003945	0.002213	7.78938	0.000000	High-High Cluster	55	68.3854
2010	Binjiang	2.520364	0.011723	0.001289	4.929564	0.000001	High-High Cluster	11	73.5799
2010	Yuhang	3.70827	0.000209	0.000700	5.100778	0.000000	High-High Cluster	62	1208.13
2010	Xihu	3.498442	0.000468	0.001454	6.977568	0.000000	High-High Cluster	53	316.974
2010	Deqing	3.929277	0.000085	−0.000266	−2.448328	0.014352	Low-High Cluster	13	975.259
2010	Daishan	−2.849547	0.004378	0.000149	3.776575	0.000159	Low-Low Cluster	0	268.933
2010	Shengsi	−2.457609	0.013987	0.000068	3.008329	0.002627	Low-Low Cluster	0	75.2655
2010	Dinghai	−2.540258	0.011077	0.000224	3.146424	0.001653	Low-Low Cluster	3	502.969
2011	Tonglu	3.503193	0.000460	0.000213	3.967588	0.000073	High-High Cluster	35	1849.72
2011	Fuyang	3.540464	0.000399	0.000370	5.06371	0.000000	High-High Cluster	71	1815.07
2011	Lin’an	3.547191	0.000389	0.000109	4.596999	0.000004	High-High Cluster	93	3108.66
2011	Yuhang	2.880442	0.003971	0.000472	3.455068	0.000550	High-High Cluster	103	1208.13
2011	Xihu	2.660141	0.007811	0.000788	3.804385	0.000142	High-High Cluster	82	316.974
2011	Daishan	−2.713581	0.006656	0.000135	3.431266	0.000601	Low-Low Cluster	0	268.933
2011	Beilun	−2.814063	0.004892	0.000266	2.896999	0.003768	Low-Low Cluster	10	549.991
2011	Dinghai	−2.862032	0.004209	0.000243	3.417995	0.000631	Low-Low Cluster	4	502.969
2012	Tonglu	4.727728	0.000002	0.000235	4.512294	0.000006	High-High Cluster	29	1849.72
2012	Fuyang	5.014653	0.000001	0.001148	16.114149	0.000000	High-High Cluster	132	1815.07
2012	Lin’an	3.756233	0.000172	0.000090	3.902807	0.000095	High-High Cluster	110	3108.66
2012	Xiacheng	3.299505	0.000969	0.001324	4.454942	0.000008	High-High Cluster	49	30.5015
2012	Shangcheng	2.971575	0.002963	0.002044	7.216213	0.000000	High-High Cluster	50	26.3608
2012	Gongshu	3.299505	0.000969	0.000834	3.048334	0.002301	High-High Cluster	41	68.3854
2012	Xihu	4.099386	0.000041	0.001891	9.35784	0.000000	High-High Cluster	105	316.974

**Table 5:** Critical indexes of ARIMA model and simple seasonal ESM.

Model	BIC	R-Square	RMSE	MAPE	MAE	MSE
ARIMA(6,1,0)	7.30	0.73	36.31	15.94	27.56	1318.56
Simple Seasonal ESM	7.11	0.78	33.04	14.25	24.63	1091.38

**Table 6:** Prediction results of 12 months in 2013 using Simple Seasonal ESM.

Time	Reported Cases	Predicted Cases 95% CI
Jan-2013	238	138.8(72.91,204.69)
Feb-2013	252	134.8(61.14,208.46)
Mar-2013	285	123.63(42.96,204.31)
Apr-2013	160	144.47(57.33,231.6)
May-2013	146	182.8(89.65,275.95)
Jun-2013	79	137.3(38.5,236.1)
Jul-2013	97	130.97(26.83,235.1)
Aug-2013	88	110.13(0.92,219.35)
Sep-2013	112	115.47(1.4,229.54)
Oct-2013	112	128.8(10.07,247.53)
Nov-2013	126	172.14(48.93,295.34)
Dec-2013	138	123.3(−4.23,250.83)

CI: confidence interval.
